# Physiological and Pathophysiological Roles of Mitochondrial Na^+^-Ca^2+^ Exchanger, NCLX, in Hearts

**DOI:** 10.3390/biom11121876

**Published:** 2021-12-14

**Authors:** Ayako Takeuchi, Satoshi Matsuoka

**Affiliations:** 1 Department of Integrative and Systems Physiology, Faculty of Medical Sciences, University of Fukui, Fukui 910-1193, Japan; smatsuok@u-fukui.ac.jp; 2 Life Science Innovation Center, University of Fukui, Fukui 910-1193, Japan

**Keywords:** mitochondria, heart, mitochondrial Na^+^-Ca^2+^ exchanger, NCLX, metabolism, Ca^2+^ signaling

## Abstract

It has been over 10 years since *SLC24A6/SLC8B1*, coding the Na^+^/Ca^2+^/Li^+^ exchanger (NCLX), was identified as the gene responsible for mitochondrial Na^+^-Ca^2+^ exchange, a major Ca^2+^ efflux system in cardiac mitochondria. This molecular identification enabled us to determine structure–function relationships, as well as physiological/pathophysiological contributions, and our understandings have dramatically increased. In this review, we provide an overview of the recent achievements in relation to NCLX, focusing especially on its heart-specific characteristics, biophysical properties, and spatial distribution in cardiomyocytes, as well as in cardiac mitochondria. In addition, we discuss the roles of NCLX in cardiac functions under physiological and pathophysiological conditions—the generation of rhythmicity, the energy metabolism, the production of reactive oxygen species, and the opening of mitochondrial permeability transition pores.

## 1. Introduction

It has been almost a half century since a mitochondrial Na^+^-Ca^2+^ exchange (NCX_mit_) system was discovered in the heart [1], and it has been more than 10 years since *SLC24A6/SLC8B1,* coding the Na^+^/Ca^2+^/Li^+^ exchanger (NCLX), was identified as the gene responsible for the system [2]. Owing to this molecular identification, our understanding of physiological and pathophysiological roles of NCX_mit_ has dramatically increased in various types of cells, including cardiomyocytes, neurons, astrocytes, B lymphocytes, pancreatic β cells, and brown adipocytes [3,4,5,6,7,8,9,10]; see also reviews [11,12,13]. In the heart, NCX_mit_ comprises the major Ca^2+^ efflux mechanism to balance against Ca^2+^ influx via mitochondrial Ca^2+^ uniport (CU_mit_) activity. For the physiological functions of cardiomyocytes, it is vital for mitochondrial matrix Ca^2+^ to be maintained within an appropriate range because several metabolic enzymes are activated by Ca^2+^ to supply ATP, meeting cellular ATP demand, but excess Ca^2+^ causes mitochondrial dysfunction via opening of mitochondrial permeability transition pores (mPTP) (see other reviews for details [14,15]). In this review, we provide an overview of recent findings regarding NCX_mit_, especially focusing on its biophysical properties, distributions, and physiological and pathophysiological roles in the heart.

## 2. Tissue-Specific Characteristics of Mitochondrial Ca^2+^ Dynamics

The mitochondrial Ca^2+^ handling system varies from tissue to tissue, which may contribute to tissue-specific tuning of mitochondrial as well as cellular functions. It is well accepted that CU_mit_ activity is lower in the heart than in other tissues [16]. This lower CU_mit_ activity may be due to differences in stoichiometry among a pore-forming protein (MCU), a dominant negative subunit (MCUb), and/or an EF-hand containing Ca^2+^-sensitive regulator (MICU1)—an MCUb:MCU ratio that is higher and an MICU1:MCU ratio that is lower in the heart [17,18,19]. In addition, Wescott et al. [20] reported that “gate-keeping” of CU_mit_ via a certain cytosolic Ca^2+^ threshold was not observed in heart, though it has been well described functionally and structurally in other tissues or cell types (see review [21]). These heart-specific characteristics of the mitochondrial Ca^2+^ influx system may contribute to preventing mitochondrial Ca^2+^ overload in the heart, where cytosolic Ca^2+^ periodically rises. 

In order to balance against the mitochondrial Ca^2+^ influx, NCX_mit_ and H^+^-Ca^2+^ exchange (HCX_mit_) extrude Ca^2+^ from mitochondria, with the former accounting for the major component in excitable tissues such as the heart and brain, and the latter being dominant in non-excitable tissues such as the liver and kidney (see review [22]). Rysted et al. [23] quantitatively compared the NCX_mit_ activity in mitochondria isolated from mouse brains, livers, and hearts. By evaluating extra-mitochondrial Ca^2+^ using Calcium Green-5N, they demonstrated that the rate of Na^+^-dependent Ca^2+^ efflux from mitochondria was ~3-fold larger in the brain than in the heart. This well agrees with the lower CU_mit_ activity in the heart compared with other tissues [16,18]. Interestingly, the NCX_mit_ activity in the liver was negligible, despite the fact that it has the highest mRNA expression level of NCLX. The authors attributed this to extra-mitochondrial expression of NCLX protein in the liver.

In the heart, the fraction of Na^+^-dependent Ca^2+^ efflux to total Ca^2+^ efflux is 60–100%, depending on species and experimental conditions [8,23,24,25]. The remaining fraction should be mediated by HCX_mit_, though its contribution in the heart has been controversial. Leucine-zipper-EF hand-containing transmembrane (Letm1), which was initially shown to mediate H^+^-dependent Ca^2+^ influx into mitochondria [26,27], was proposed as the gene responsible for HCX_mit_. Natarajan et al. [28] detected H^+^-induced Ca^2+^ efflux from rat cardiac mitochondria, which were dependent on the free matrix Ca^2+^ concentration. Furthermore, they confirmed Letm1-mediated Ca^2+^ efflux from mitochondria by demonstrating a diminished Ca^2+^ efflux rate in permeabilized H9c2 cells due to Letm1 knockdown. Interestingly, they found that the expression level of the Letm1 protein in mitochondria was higher in the heart than in the liver, though the functional contribution of HCX_mit_ was much higher in the liver than in the heart. Post-translational modifications or extra-mitochondrial localization of Letm1 protein in the heart, just as reported for NCLX [23], may explain the disparity between the expression level and function.

## 3. Biophysical Properties of NCX_mit_

The electrogenicity of NCX_mit_ had been controversial [29,30,31,32]. These controversies were raised largely because mitochondrial membrane potential (∆Ψ) is affected by H^+^ movements across the mitochondrial inner membrane via the electron transport chain, F_1_F_o_-ATP synthase, and so on. Therefore, detecting an NCX_mit_-mediated ∆Ψ change with the exclusion of the ∆Ψ change via the fluctuation of energy metabolism was challenging. In order to overcome this problem, it is necessary to clamp ∆Ψ. Recently, our group succeeded in recording membrane currents through NCX_mit_ in mouse cardiac mitochondria using whole-mitoplast patch clamp methods, thus settling the controversy [8].

**Figure 1:**
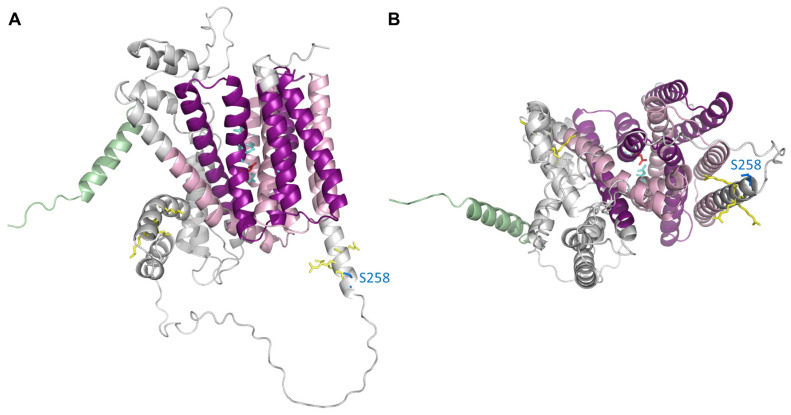
Putative three-dimensional structure of human NCLX (UniProtKB accession number Q6J4K2) predicted using AlfaFold [36]. The pdb file (AF-Q6J4K2-F1-model_v1) was downloaded from the AlphaFold Protein Structure Database (https://alphafold.ebi.ac.uk/ accessed on 05 November 2021) and graphics were prepared using PyMOL v.2.1.0. (**A**) Side view, (**B**) bottom view. Putative mitochondria transit peptide and two sodium/calcium exchanger membrane regions are shown in green and pale and dark pink, respectively. Putative protein kinase A (PKA) phosphorylation site, S258 [39], is shown as blue sticks. Putative amino acids rendering Li^+^ selectivity, Na^+^ selectivity [34], and those sensitive to ∆Ψ depolarization [38] are shown as light blue, red, and yellow sticks, respectively.

The characteristics of the NCX_mit_ current in forward mode, i.e., an extra-mitochondrial Na^+^-induced inward current with Ca^2+^ in the pipette, corresponded well to those in previous reports—the currents were diminished by the NCX_mit_ inhibitor CGP-37157 [32,33], the Hill coefficient for cytosolic Na^+^ was around 3–4 [29,32], and Li^+^ can be substitutable for Na^+^ with ~70% lower efficacy [23,24]. One deviation was that the half-maximum concentration for Na^+^, 35.6 mM, was higher than the reported value of 1–8 mM in the heart (8 mM in [29]; 1 mM in [32]), possibly because the Na^+^-permeable background current could exist in the mitoplast preparations. The lower efficacy of Li^+^ compared to Na^+^ in exchanging for Ca^2+^ was also reported in brain mitochondria, to a similar extent as in the heart [10,23]. Through molecular modeling analysis based on *Metanococcus jannaschii* NCX_Mj and *Archaeoglobus fulgidus* CAX_Af, combined with functional analysis of human NCLX mutants, distinct amino acid residues in NCLX were identified as determining Na^+^ or Li^+^ binding [34]. That is, N149, P152, D153, N467, S468, and G494 were proposed to render Li^+^ selectivity, whereas D471 was proposed to render Na^+^ selectivity [34]. Giladi et al. [35] independently analyzed NCX_Mj-derived mutant NCLX_Mj, with nine substituted resides causing a NCLX-like phenotype, and found that peptides 248–255 were sensitive only to Li^+^ binding, but not to Na^+^ nor Ca^2+^ binding. Therefore, it is reasonable that the efficacy of exchanging for Ca^2+^ was different between Na^+^ and Li^+^. Although the three-dimensional (3D) structure of NCLX has not been solved yet, recent advances in artificial intelligence-based structure prediction methods makes it possible to easily visualize a putative 3D structure of NCLX. Figure 1 shows a putative 3D structure of human NCLX (Q6J4K2), predicted using AlphaFold [36], with specific residues highlighted that are suggested to be functionally important.

In the whole-mitoplast patch clamp experiments, the NCX_mit_ current in reverse mode—an extra-mitochondrial Ca^2+^-induced outward current with Na^+^ in the pipette—could not be recorded [8]. This was rather surprising to us because the reverse mode of NCX_mit_ activity was previously reported to exist in mitochondria of rat cardiomyocytes [32,37]. Further evaluation of intra-mitochondrial Ca^2+^ using Fluo-8 in isolated mitochondria revealed that the reverse mode of NCX_mit_ activity did exist in the heart. That is, CGP-37157-sensitive and intra-mitochondrial Na^+^-dependent Ca^2+^ influx was detected, but the rate was too slow to be recorded electrophysiologically [8]. What is the mechanism underlying the slow NCX_mit_ activity in reverse mode? One possible explanation may be an allosteric regulation of NCLX by ∆Ψ, as reported in SH-SY5Y neuronal cells and in HEK-293T cells [38]. The authors showed that mild ∆Ψ depolarization inhibited NCX_mit_ via two clusters of positively charged residues, which are putatively located in the regulatory loop around the inner membrane (yellow sticks in Figure 1). They also showed that phosphorylation of S258 in human NCLX, known to be a protein kinase A (PKA) target site [39] (blue sticks in Figure 1), could override the regulation. Since mitoplasts and isolated mitochondria were free of cytosolic ingredients, it could be possible that phosphorylation at the residue was not sufficient to override the depolarization-mediated inhibition under the experimental conditions of [8]. The unfavorable reversal of NCX_mit_ was also reported in leukotriene C_4_-stimulated mast cells with depolarized mitochondria [40]. Interestingly, however, mitochondrial fusion protein mitofusin (MFN) 2 knockdown caused repetitive reversal of NCX_mit_ even under depolarized conditions, resulting in mitochondrial and cytosolic Ca^2+^ oscillation. It is worth examining phosphorylation status at NCLX S258 in MFN2-knockdown cells.

In dopaminergic neurons, it was demonstrated that PTEN-induced putative kinase 1 (PINK1) at mitochondria activated PKA, thereby phosphorylating S258 of NCLX [39]. PINK1 deficiency is closely associated with mitochondrial abnormalities and the progression of early-onset familial Parkinson’s disease [41,42]. In addition, recent studies demonstrated deficiencies of PINK1 and mitochondrial function in failing hearts, such as in hearts with late stages of dystrophic cardiomyopathy and sepsis [43,44]. Abnormal NCX_mit_ activity via PINK1 deficiency may be associated with mitochondrial dysfunction in these failing hearts.

## 4. Spatial Distribution of NCX_mit_ in Cardiomyocytes

Mitochondria are physically and electrically connected with each other via intermitochondrial junctions and form “mitochondrial reticulum” throughout the cell in the skeletal and cardiac muscles [45,46]. However, there is still functional heterogeneity depending on their spatial distributions—one just beneath the sarcolemmal membrane (subsarcolemmal mitochondria, SSM), one between myofibrils (interfibrillar mitochondria, IFN), and one near the nucleus (perinuclear mitochondria, PNM). This heterogeneity may contribute to dealing with region-specific energy demands, sensitivity to oxidative stress, Ca^2+^ handling, and so on [47,48].

Using mitochondrial Ca^2+^ indicator Myticam-expressing rabbit cardiomyocytes, Lu et al. [49] demonstrated that 1 Hz electrical stimulation induced faster Ca^2+^ uptake in IFM than in PNM, whereas the post-stimulation Ca^2+^ efflux was comparable. The higher uptake but comparable efflux of mitochondrial Ca^2+^ in IFM than in PNM resulted in the higher sensitivity to phenylarsine oxide for the opening of mPTP, shown as faster ∆Ψ depolarization. In addition, the spatial difference of CU_mit_ activity was preserved in permeabilized cardiomyocytes under the conditions of a clamped cytosolic Ca^2+^ concentration with a disabled sarcoplasmic reticulum (SR) Ca^2+^ pump (SERCA), but the efficacy was lower than that observed in intact cardiomyocytes. Therefore, an intrinsic difference in CU_mit_ activity may be further amplified by local SR Ca^2+^ release-associated excitation–contraction coupling in intact cardiomyocytes.

While Ca^2+^ efflux activity was comparable in IFM and PNM [49], it was shown to be larger in SSM than in IFM [50]. Immunofluorescence analyses using stochastic optical reconstruction microscopy revealed that NCLX localized mainly in SSM near (<20 nm) voltage-dependent Na^+^ channel Na_v_1.5 clusters, and hardly existed in IFM of rabbit ventricular myocytes. Functional analyses showed that the treatment of cells with a Na^+^ channel blocker, tetrodotoxin, increased intensity of Rhod-2, an indicator of mitochondrial Ca^2+^, in SSM but not in IFM. Since NCX_mit_ inhibition by CGP-37157 produced similar results as those obtained using tetrodotoxin, it was suggested that Na^+^ accumulation at the subsarcolemmal space via Na_v_1.5 potentiated NCLX-mediated Ca^2+^ efflux from mitochondria, preventing mitochondrial Ca^2+^ accumulation. The authors further examined the contribution of the physical and functional coupling of Na_v_1.5-NCLX to reactive oxygen species (ROS) production in mitochondria, which is closely associated with mitochondrial Ca^2+^, as will be described in Section 5.3. However, they failed to detect spatial differences in mitochondrial ROS production, possibly due to the propagation of ROS via the mitochondrial reticulum [45,46,51]. Further analyses are necessary to prove the physiological and pathophysiological roles of this Na_v_1.5–NCLX coupling. Quantitative analysis of spatial Na^+^ regulation via the Na^+^-K^+^ ATPase and Na^+^ channel, as performed by Skogestad et al. [52] would help in understanding this subject.

In addition to the heterogeneities of mitochondrial Ca^2+^ dynamics at differentially localized cardiac mitochondria, intra-mitochondrial heterogeneities were also reported. Lu et al. [53] evaluated mitochondrial Ca^2+^ transients using Myticam-expressing rat ventricular myocytes. They analyzed 0.2 Hz stimulation-induced small mitochondrial Ca^2+^ transients, which increased from ~150 nM by ~30 nM and found that the upstroke was faster at position near the Z-line than near the M-line but the decay was comparable. Although MCU immunofluorescence showed a uniform distribution over the mitochondrion, it was suggested that CU_mit_ activity was higher at mitochondria facing junctional SR (jSR) than at those facing bulk cytosol, whereas Ca^2+^ efflux activity was comparable. This is reasonable because mitochondria–jSR association creates high Ca^2+^ microdomains near the dyadic space, which enables them to meet the low affinity of CU_mit_ for Ca^2+^ uptake (see review [54]). 

De La Fuente et al. [55,56] further explored the spatial heterogeneities of mitochondrial Ca^2+^ dynamics. Using conventional and super-resolution immunofluorescence analyses of isolated cardiac mitochondria and isolated cardiomyocytes, they demonstrated that about 50% of MCU were closely co-localized with the SR Ca^2+^ release channel ryanodine receptor (RyR) 2 [55]. The authors explained that the divergence of this biased MCU distribution from the previously reported uniform distribution [53] was attributable to the antibodies chosen, since one used in [53] gave non-specific signals in MCU knockout mouse-derived cardiomyocytes. Supporting the idea of MCU-RyR2 colocalization, MCU and EMRE, which are essential CU_mit_ regulator proteins, were more abundant in crude mitochondria than in Percoll-purified mitochondria, and were also found in jSR [55]. On the other hand, the NCLX protein was more abundant in pure mitochondria than in crude mitochondria, and was not found in jSR [56]. Moreover, the authors strengthened their findings on distinct distributions of MCU and NCLX by means of functional assays. CU_mit_ activity—CU_mit_ inhibitor Ru360-sensitive ^45^Ca^2+^ uptake corrected with citrate synthase activity—was much higher in isolated jSR than that in isolated mitochondria. On the contrary, ^45^Ca^2+^ retention assays revealed that Na^+^- and CGP-37157-sensitive mitochondrial Ca^2+^ efflux activity was much higher in pure mitochondria than that in jSR. This ^45^Ca^2+^ efflux activity became larger and smaller in heart-specific NCLX overexpressing and knockout mice, respectively. The authors proposed that the spatially separated distribution of MCU-RyR2 and NCLX contributes to minimizing the energy cost for maintaining ∆Ψ. In other word, if MCU-RyR2 were near NCLX, ∆Ψ would depolarize both due to Ca^2+^ influx via CU_mit_ and due to Ca^2+^ efflux via NCX_mit_. Accordingly, the spatial separation of MCU-RyR2 and NCLX should be necessary for optimizing mitochondrial Ca^2+^ signals and energy cost. Interestingly, it was demonstrated that NCLX efficiently supplies Ca^2+^ from mitochondria to the SR/endoplasmic reticulum (ER) via SERCA, thereby regulating the automaticity of HL-1 cardiomyocytes, as well as antigen receptor-mediated Ca^2+^ signaling of B lymphocytes [3,5]. It is worth evaluating the physical coupling of NCLX and SERCA in cardiomyocytes, which would fill in the last piece in our understanding of the efficient Ca^2+^ cycling between SR and mitochondria.

## 5. Role of NCX_mit_ in Cardiomyocyte Functions

### 5.1. Role of NCX_mit_ in Cardiac Rhythmicity

Since CU_mit_ comprises only 1–2% of total Ca^2+^ removal from cardiomyocytes [53,57,58], the contribution of NCX_mit_ to cytosolic Ca^2+^ transients and to action potential generation has been considered negligible. However, in a spontaneously beating cell line, HL-1, originating from mouse atrial myocytes, NCLX knockdown by siRNA significantly decelerated the upstrokes of action potentials and Ca^2+^ transients, and prolonged the cycle lengths [5]. Consistently with the results of other studies [53,57,58], NCLX knockdown did not change the rest and peak fluorescence ratios of Indo-1, an indicator of the cytosolic Ca^2+^ level. Rather, it was demonstrated that NCLX knockdown decreased caffeine-responsive SR Ca^2+^ content and slowed subsequent SR Ca^2+^ reuptake rate, evaluated using a FRET protein Cameleon D1ER. Further analyses of a mathematical model combined with experimental validation revealed that (1) the automaticity of HL-1 cells was driven by the so-called “Ca^2+^ clock” mechanism, in which a Ca^2+^ leak from SR potentiates the inward current via sarcolemmal Na^+^-Ca^2+^ exchange to facilitate membrane depolarization, (2) NCLX knockdown decreased the Ca^2+^ supply from mitochondria to SR, thereby decelerating SR Ca^2+^ leak, delaying the activation of the inward current through sarcolemmal Na^+^-Ca^2+^ exchange, and thus delaying the activations of voltage-dependent Na^+^ and Ca^2+^ currents, causing a cycle length prolongation.

Considering that HL-1 cells are derived from atrial myocytes, which are quiescent under physiological conditions, NCX_mit_ may be involved in abnormal automaticity of atria, such as atrial flutter and atrial ectopic tachycardia. In addition, it may also be plausible that abnormal NCX_mit_ function causes ventricular arrhythmias. In fact, the involvement of abnormal NCX_mit_ activity in altered rhythmicity was suggested in mouse embryonic stem cell-derived as well as in human induced pluripotent stem cell-derived ventricular myocytes, where the “Ca^2+^ clock” drives the automaticity [59]. In addition, arrhythmic evens with QRS interval widening were observed in tamoxifen-induced heart-specific conditional NCLX-knockout mice, though the events only occurred immediately before death [25].

The question of whether NCX_mit_ participates in the automaticity of normal pacemaker cells, i.e., sinoatrial (SA) node cells, is still a big issue. The automaticity of SA node cells has been proposed to be driven by a “coupled-clock” pacemaking system, which is composed of a sarcolemmal ion channel/transporter-derived rhythm (“membrane clock”) and subsarcolemmal Ca^2+^ release (LCR)-related rhythm (“Ca^2+^ clock”) [60,61,62]. In the former, pacemaker channels such as the hyperpolarization-activated cation channel and various other inward membrane currents at the plasma membrane drive diastolic depolarization. In the latter, LCR from SR activates the inward current via sarcolemmal Na^+^-Ca^2+^ exchange to drive diastolic depolarization. NCX_mit_ may modulate the “Ca^2+^ clock” part in SA node cells, as observed in HL-1 cells [5]. In fact, application of an NCX_mit_ inhibitor, CGP-37157, slowed the firing rate of rabbit as well as mouse SA node cells [63,64]. However, recent imaging studies of mouse SA node preparations revealed marked heterogeneity of LCR and action potential-induced Ca^2+^ transients within and among SA node cells [65]. That is, some SA node cells generated only LCR and did not fire; some only generated action potential-induced Ca^2+^ transients and did not generate LCR; and some generated LCR during the diastolic phase before an occurrence of action potential-induced Ca^2+^ transients. These data suggest that the coupling degree of the “coupled-clock” system may differ among SA node cells in vivo. Our model analyses suggested that NCX_mit_ reduction in an SA node cell which is solely driven by the “membrane clock” accelerates, instead of decelerating, the firing rate [11]. NCX_mit_ reduction-mediated slowing of automaticity in “Ca^2+^ clock”-driven cells may be compromised by NCX_mit_-mediated acceleration of automaticity in “membrane clock” cells in the SA node region. In fact, tamoxifen-induced NCLX deletion in the adult mouse heart, with a 70% reduction of NCLX protein 3 days after tamoxifen treatment, did not show altered sinus rhythms except for on the date of death, 8–10 days after tamoxifen treatment [25]. In vivo imaging of the SA node of NCLX-knockout mice would clarify the quantitative roles of NCX_mit_ in pacemaking activity.

### 5.2. Role of NCX_mit_ in Cardiac Energetics

**Figure 2:**
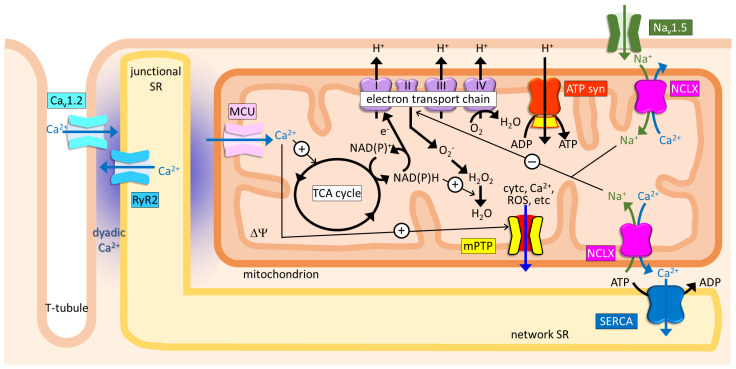
An overview of the NCLX-mediated physiological and pathophysiological functions in a cardiomyocyte. ATPsyn, F_1_F_o_-ATP synthase; Ca_v_1.2, L-type Ca^2+^ channel; cytc, cytochrome c; ∆Ψ, mitochondrial membrane potential; MCU, mitochondrial Ca^2+^ uniporter complex; mPTP, mitochondrial permeability transition pores; Na_v_1.5, voltage-dependent Na^+^ channel; ROS, reactive oxygen species; RyR2, ryanodine receptor 2; SERCA, sarcoplasmic reticulum Ca^2+^ pump; SR, sarcoplasmic reticulum; TCA, tricarboxylic acid.

The heart is continuously pumping blood around the body, which is energetically driven by ATP hydrolysis. In the healthy adult heart, ATP synthesis is mainly dependent on mitochondrial oxidative phosphorylation, and the process is strictly regulated to balance the large, dynamically changing energy demands [66]. One candidate factor for the regulation is Ca^2+^, which activates three mitochondrial dehydrogenases—pyruvate dehydrogenase complex (PDHC), isocitrate dehydrogenase, and 2-oxoglutarate dehydrogenase (OGDH) [67] (Figure 2). The product NADH is oxidized in the electron transport chain, causing the proton motive force to be utilized for ATP synthesis via F_1_F_o_ ATP synthase. Therefore, the contribution of NCX_mit_, as one of the determinants of the mitochondrial Ca^2+^ level, to cardiac energetics has been an issue to be clarified.

This was first shown in whole-cell patch clamp experiments using guinea pig ventricular myocytes loaded with Rhod-2 for evaluating mitochondrial Ca^2+^ changes [68]. It was demonstrated that when NCX_mit_ became more active with 15 mM compared with 5 mM Na^+^ in the pipette, the mitochondrial Ca^2+^ increase induced by an abrupt workload increase (3–4 Hz pacing in the presence of isoproterenol) was diminished. At the same time, under the condition of 15 mM Na^+^ in the pipette, NADH autofluorescence decreased upon the workload increase, indicating that mitochondrial Ca^2+^ was not sufficient enough to activate NADH production by mitochondrial dehydrogenases. An NCX_mit_ inhibitor, CGP-37157, restored the workload-induced Ca^2+^ accumulation in mitochondria and attenuated the NADH decrease [69]. Since a cytosolic Na^+^ increase and energy starvation are characteristic properties of failing heart [70], the authors further studied a guinea pig model of heart failure which was induced by aortic constriction with/without β-adrenergic receptor stimulation [69,71]. In ventricular myocytes from failing hearts, where cytosolic Na^+^ evaluated from SBFI ratio image was ~15 mM compared to ~5 mM in sham myocytes, the abrupt workload increase caused essentially the same responses of mitochondrial Ca^2+^ (Rhod-2 or Myticam) and NADH as those reported with 15 mM Na^+^ in the pipette [68,69]—the diminished increase of mitochondrial Ca^2+^ and the subsequent NADH starvation upon the workload increase. More importantly, the changes were restored in the presence of an NCX_mit_ inhibitor, CGP-37157, to levels similar to those observed in sham myocytes. These results suggested a causative role of NCX_mit_ in the energy starvation of the failing heart. In addition, as will be explained in Section 5.3, chronic treatment of the animals with CGP-37157 partially prevented cardiac dysfunctions. Accordingly, the authors proposed that blocking of NCX_mit_ is a novel strategy for treating heart failure [71].

However, the contribution of NCX_mit_ to cardiac energetics in the failing heart may not be as large as that expected from experiments using cardiomyocytes, where an extreme workload change was applied—rapid 3–4 Hz pacing from a quiescent state, which hearts in situ never experience [68,69,71]. Recently, the effects of chronic and acute myocardial Na^+^ loads on cardiac energetics were extensively studied in Langendorff-perfused mouse hearts with ^23^Na, ^31^P, ^13^C NMR, and ^1^H-NMR metabolomic profiling [72]. Chronic (phospholemman PLM^3SA^ mouse) and acute (treatment with ouabain and blebbistatin) inhibition of Na^+^-K^+^ ATPase, as well as pressure-overload-induced cardiac hypertrophy caused a cytosolic Na^+^ increase, and switched the substrate preference from fatty acid to carbohydrate oxidation, which are characteristic features frequently observed in failing hearts [70,73]. The acute Na^+^ elevation resulted in the most severe metabolic alterations, such as decreased metabolite levels of tricarboxylic acid (TCA) cycle intermediates downstream from OGDH (succinate, fumarate, and malate), suggesting the reduced Ca^2+^-dependent activation of TCA cycle dehydrogenases. However, regardless of the strategy for cytosolic Na^+^ elevations, the energy supply was maintained, as is evident from the preserved ATP, phosphocreatine (PCr), PCr/ATP ratio, NADH, and pH. Metabolome profiles obtained with NMR, as well as in silico predictions using CardiNet, revealed that they were achieved at the cost of extensive metabolic flux remodeling. Therefore, the impact of impaired cytosolic Na^+^ homeostasis on mitochondrial ATP production should be mechanistically more complex than what has been suggested in isolated cardiomyocytes. In all three sets of hearts with elevated cytosolic Na^+^, treatment with CGP-37157 reversed the substrate preference from carbohydrate to fatty acid oxidation with normalized levels of the depleted metabolites. This suggests a therapeutic potential for CGP-37157 in the treatment of the metabolic reprograming that occurs before energetic impairments.

To the contrary, detrimental contributions of NCX_mit_ to cardiac energetics were not suggested in heart-specific NCLX-overexpression mice [25]. There were no apparent differences between control and NCLX-overexpression mice’s ventricular myocytes in terms of the NAD^+^/NADH ratio, oxidative phosphorylation evaluated by seahorse analyses with either pyruvate or palmitate as energy substrates (basal, ATP-linked and maximum respirations, spare capacity, and proton leak), nor in the phosphorylation level of mitochondrial Ca^2+^-responsive PDHC. These findings suggested that NCLX overexpression had marginal effects on cardiac energetics. Rather, as will be explained in Section 5.3, NCLX overexpression prevented the cardiac dysfunctions of ischemia-reperfusion injury and ischemic heart failure. It should be noted that the basal mitochondrial Ca^2+^ level, evaluated as carbonyl cyanide 4-(trifluoromethoxy)phenylhydrazone)-responsive Fura-2 intensity, was comparable between control and NCLX-overexpressing cardiomyocytes, indicating that NCLX overexpression did not cause excessive deprivation of mitochondrial Ca^2+^, in spite of an increase in mitochondrial Ca^2+^ efflux rate by 88%. Our model analyses suggested that cytosolic Ca^2+^ within its physiological range, 100 nM–2 μM, does not largely affect steady-state levels of energy substrates, though a lower cytosolic Ca^2+^ level collapsed the system because of mitochondrial Ca^2+^ deprivation [74,75]. Therefore, some compensation or backup mechanisms may work to prevent mitochondrial Ca^2+^ deprivation via NCLX overexpression. It would be informative to further evaluate the mitochondrial Ca^2+^ level, cytosolic Na^+^ level, and metabolome profiles in failing hearts with or without NCLX overexpression.

### 5.3. Role of NCX_mit_ in ROS Production and mPTP Opening 

Mitochondria are a major source of ROS, the production of which is tightly coupled with ATP synthesis—Complex I and Complex III in the electron transport chain produce O_2_^−^ from O_2_ oxidation. Then, manganese-dependent superoxide dismutase converts O_2_^−^ to H_2_O_2_, which is eliminated by antioxidant scavenge systems, such as glutathione peroxidase and peroxiredoxin [76] (Figure 2). Excessive amounts of ROS, either via overproduction or via reduced scavenging pathways, exert detrimental effects on mitochondrial function, such as uncoupling of the electron transport chain to reduce ATP production, and triggering mPTP opening by sensitizing mPTP to mitochondrial Ca^2+^. mPTP opening causes a burst of ROS released from mitochondria, in a process named ROS-induced ROS release, which impairs excitation–contraction coupling via modulating multiple ion channels and transporters, as well as via chronic remodeling [77,78] (Figure 2). Based on the fact that mitochondrial Ca^2+^ activates three dehydrogenases and the product NADH promotes ROS by-production via the electron transport chain, and that mitochondrial Ca^2+^ overload is a key trigger for mPTP opening, followed by a burst ROS release, altered mitochondrial Ca^2+^ dynamics have been implicated to be closely associated with ROS dynamics in failing and injured hearts [79,80].

Hamilton et al. [81] demonstrated the involvement of NCX_mit_ in ROS production, SR Ca^2+^ handling, and arrhythmogenesis in rat ventricular myocytes. They monitored mitochondrial Ca^2+^ using a biosensor mtRCamp1h, and showed that NCX_mit_ inhibition by CGP-37157 decelerated mitochondrial Ca^2+^ decay, thereby enhancing mitochondrial Ca^2+^ accumulation triggered by 2 Hz electrical stimulation in the presence of isoproterenol. This resulted in larger ∆Ψ depolarization monitored by TMRM, increased ROS in the mitochondria-SR microdomain evaluated using ER-tuned redox sensor ERroGFP_iE, increased RyR oxidation as evident from increased immunoprecipitation with anti-dinitrophenyl-antibody, and increased proarrhythmic Ca^2+^ waves. The authors also showed that this cascade further exacerbated proarrhythmic-triggered activity in hypertrophied hearts, which were induced by thoracic aortic banding. 

The detrimental consequences of NCX_mit_ inhibition were more prominent in NCLX-knockout mice. The germline NCLX knockout was unsuccessful, and adult acute heart-specific NCLX knockout, in which NCLX protein expression was reduced by ~70%, caused ~87% lethality within 2 weeks due to severe myocardial dysfunction accompanying increased ROS, evaluated with dihydroethidium and MitoSox red, and mitochondrial swelling [25]. This lethality was attributable to mitochondrial Ca^2+^ overload-mediated mPTP opening, because the depletion of the mPTP component cyclophilin D on the NCLX conditional knockout background rescued the myocardial dysfunction and lethality following tamoxifen-induced NCLX ablation. Those authors suggested that NCLX-mediated Ca^2+^ efflux was necessary to maintain an appropriate mitochondrial Ca^2+^ level, which was vital for preventing mPTP opening and excessive ROS production, and for survival. The idea was further confirmed in heart-specific NCLX-overexpression mice subjected to ischemia-reperfusion [25]. Accordingly, NCLX overexpression reduced the ROS level evaluated using dihydroethidium in hearts with 40 min-left coronary artery ligation followed by 24 h reperfusion, and tended to decrease it 4 weeks after permanent occlusion of the left coronary artery. In addition, cardiac dysfunctions characterized by TUNEL-positive interstitial cells, fibrosis, and contractile dysfunction were all improved by NCLX overexpression. The above findings clearly indicated beneficial contributions of NCX_mit_ in ischemia-induced failing hearts.

However, a contradictory mechanism was proposed by Liu et al. [71]. As explained in Section 5.2, an abrupt workload increase resulted in a diminished increase in mitochondrial Ca^2+^, followed by NADH starvation, in failing ventricular myocytes, possibly because elevated cytosolic Na^+^ excessively extruded Ca^2+^ from mitochondria via NCX_mit_ [71]. Interestingly, dichlorodihydrofluorescein diacetate oxidation, an index of the ROS level, was dramatically increased upon an abrupt workload increase in the failing cardiomyocytes but not in the sham cardiomyocytes and this ROS production was completely diminished in the presence of an NCX_mit_ inhibitor, CGP-37157. Moreover, chronic treatment of the animals with CGP-37157 using an osmotic pump partially prevented the animals from developing heart failure, as evident from improved hypertrophic remodeling, interstitial fibrosis, contractile dysfunction, and occurrence of arrhythmia. The authors attributed the mechanism to reduced ROS scavenging capacity due to the reduced NAD(P)H levels in failing cardiomyocytes. Accordingly, these findings indicated a detrimental contribution of NCX_mit_ in failing hearts.

The abovementioned contradictory roles of NCX_mit_ in failing hearts suggested that mitochondrial Ca^2+^ did not simply correlate with ROS production. Recently, a brand-new mechanism underlying hypoxia-induced ROS production via NCX_mit_ was proposed—Na^+^–phospholipid interaction-mediated ROS regulation [82] (Figure 2). The authors first confirmed that NCX_mit_ was involved in hypoxia-induced ROS production in primary bovine aortic endothelial cells and mouse embryonic fibroblasts. Pharmacological inhibition with CGP-37157 or genetical reduction (siRNA or knockout) of NCLX diminished the cytosolic Ca^2+^ increase and cytosolic Na^+^ decrease, attenuated the reduction of the inner mitochondrial membrane fluidity and the mitochondrial ROS production caused by exposure of the cells to hypoxia (exposure of the cells to 1% O_2_). Then, the authors showed that hypoxia-induced matrix acidification via Complex I inhibition caused Ca^2+^ solubilization from calcium phosphate precipitation in the matrix, as evident from morphological (electron microscopy images) as well as from functional assays (measurements of free mitochondrial Ca^2+^ in isolated mitochondria as well as in cells). Since a mitochondrial free Ca^2+^ increase enables NCX_mit_ to extrude Ca^2+^ in exchange for Na^+^, the authors then focused on the roles of matrix Na^+^ on electron transport chains and found that only Complex II-dependent respirations were decreased by Na^+^, which was NCX_mit_-dependent, resulting in increased ROS production. The authors filled in the final piece by showing that Na^+^ directly bound to the phospholipid bilayer, as evident from infrared spectroscopy, which reduced the fluidity of the inner mitochondrial membrane for ubiquinone diffusion in the inner mitochondrial membrane, increasing the ROS production. Taken together, these findings clarified a distinct scheme of ROS production—regulation by matrix Na^+^ via NCX_mit_—from those proposed in previous reports.

## 6. Future Perspectives 

As has been described so far, knowledge on the biophysical properties, distributions, and the physiological and pathophysiological significance of NCX_mit_ in the heart is rapidly increasing. The more knowledge is accumulated, the more complicated systems are elucidated, sometimes introducing difficulties into our understanding as a whole. Taking ROS dynamics under pathological conditions as an example, some experimental evidence supports the roles of NCX_mit_ in increasing ROS production [82], whereas others support its preventive roles in relation to ROS increases [25,81]. NCX_mit_ directly modulates and is affected by cytosolic and mitochondrial concentrations of Na^+^ and Ca^2+^ ions, and ∆Ψ, which are associated with ROS balance regulation via different pathways (Figure 2). Therefore, differences in ionic conditions and mitochondrial viability under different experimental conditions or diseased states would result in different contributions of NCX_mit_.

In order to understand these complicated networks, the integration of NCX_mit_ activity and cellular/mitochondrial functions with mathematical modeling could be a powerful tool. Very recently, Cortassa et al. [83] succeeded in reconciling the apparently paradoxical roles of NCX_mit_ in ROS dynamics (see details [83]). In brief, they built two scenarios, the “Na^+^-driven oxidized scenario” and the “Ca^2+^-driven reduced scenario”, and demonstrated that variations in redox status, cytoplasmic Na^+^ concentrations and energetic capacity resulted in different mitochondrial Ca^2+^ levels and bioenergetic responses driving ATP supply and oxidative stress. The former scenario could be represented by heart failure with a reduced ejection fraction (HFrEF) in which considerable cytosolic Na^+^ overload occurs, and the latter by heart failure with a preserved or moderate ejection fraction (HFpEF, HFmEF) in which only a modest Na^+^ increase is expected. Integrating the model of matrix Ca^2+^ solubilization and precipitation from and to calcium phosphate [84] into Cortassa’s model [83] would further facilitate our understandings in this area.

The discrepancies in experimental findings obtained from isolated mitochondria, isolated cardiomyocytes, and whole hearts are other issues that remain to be solved. Recent advances in imaging techniques used to evaluate electrophysiological and metabolic properties of single cells and even organelles in tissue are promising [65,85]. By utilizing these techniques, it is expected that our understandings of the roles of NCX_mit_ in healthy as well as in failing hearts will be further deepened.

## Data Availability

Not applicable.
